# Four-Port Compact Metamaterial MIMO Antenna with Stub-Based Bandwidth Improvement

**DOI:** 10.3390/ma19081550

**Published:** 2026-04-13

**Authors:** Atziri Amaya Vargas-Balderas, José Alfredo Tirado-Méndez, Roberto Linares-Miranda, Hildeberto Jardón-Aguilar, Ruben Flores-Leal

**Affiliations:** 1 Instituto Politécnico Nacional, SEPI-ESIME Zacatenco, Av. IPN S/N, San Pedro Zacatenco, Mexico City 07300, Mexico; avargasb1400@alumno.ipn.mx (A.A.V.-B.); rlinaresy@ipn.mx (R.L.-M.); 2 CINVESTAV-IPN, Telecommunications Section, Av. IPN 2508, San Pedro Zacatenco, Mexico City 07360, Mexico; hjardon@cinvestav.mx (H.J.-A.); rfleal@cinvestav.mx (R.F.-L.)

**Keywords:** MIMO antenna, metamaterial, octagonal SRR, stub loading, 4-elements, high isolation, compact antenna

## Abstract

This paper presents the design of a compact four-element MIMO antenna based on a metamaterial structure and a reactive load generated by an open-circuit stub. The radiator array, arranged in an axial symmetry configuration, provides high inter-element isolation despite a sub-millimeter separation. The design is optimized for 5G n77/n78 band applications and employs a metamaterial structure composed of embedded octagonal split-ring resonators (SRRs) integrated on a Duroid RT5880 0500 ($\epsilon_{r}=2.2,h=1.27\;mm$) substrate. This configuration achieves high miniaturization, with individual radiators of $19\times 9.53\;mm^{2}$. Furthermore, through a stub-loading technique, the array is enhanced in two significant aspects: (a) it exhibits an increased impedance bandwidth, rising from a 23% fractional bandwidth in the stub-less design to 39% in the final architecture; and (b) a shift of the lower cut-off frequency toward lower values is obtained, resulting in a reduction of the radiator’s electrical length, which translates into physical size diminution. The total array has a size of only $28.8\times 28.8\;mm^{2}$ ($0.24\lambda_{0}\times 0.24\lambda_{0}$, considering the lower cut-off frequency). Despite the proximity between radiators and the absence of electromagnetic decoupling structures, the design ensures inter-element isolation exceeding 15 dB in the lower band and reaching values above 20 dB in the mid and upper bands. Diversity metric analysis confirms high performance, yielding an Envelope Correlation Coefficient (*ECC*) ≪0.005, Diversity Gain (*DG*) close to the ideal value (≥9.9), Total Active Reflection Coefficient (*TARC*) below −10 dB (converging in random phase analysis), and a Channel Capacity Loss (*CCL*) of less than 0.4 bits/s/Hz. Therefore, the proposed antenna stands as an ideal design for compact 5G communication devices.

## 1. Introduction

The surge of 5G services has intensified the demand for high-density antenna systems, such as Multiple-Input Multiple-Output (MIMO) antennas. This trend has driven research toward extreme miniaturization techniques that do not compromise array performance. The n77 (3.3–4.2 GHz) and n78 (3.3–3.8 GHz) bands are essential for 5G applications, requiring the design of radiators capable of operating within these ranges while occupying minimal physical space. However, antenna miniaturization typically imposes negative trade-offs on bandwidth (BW), gain, efficiency, and polarization purity [1].

Various techniques for MIMO antenna size reduction have been reported in the literature. For instance, in [2], a C-shaped ground plane was implemented to introduce multiple resonances and optimize impedance matching alongside miniaturization. In [3], the use of split-ring resonator (SRR)-based metamaterials enabled a 50% size reduction compared to conventional disc monopoles. Similarly, in [4], the radiator geometry was modified using slots to alter surface current paths, integrating a Defected Ground Structure (*DGS*) technique to tune the system’s distributed capacitance and inductance.

Building on these precedents, this work employs material-loaded antenna techniques [1] for the design, which induce wave slowing proportional to the equation $1/\sqrt{\varepsilon_{r}\mu_{r}}$ [5]. Among these techniques, the implementation of metamaterial structures, particularly SRRs, stands out. These structures allow for the manipulation of constitutive parameters (*μ* and *ε*) to achieve electromagnetic wave propagation without diffraction within the material, thereby reducing the electrical path [6,7].

Nonetheless, miniaturized antennas often suffer from bandwidth limitations, restricting their applicability in communication systems. To mitigate this constraint, this work employs a compensation technique via stub loading. This method facilitates the redistribution of surface currents, either by increasing the electrical path or by introducing a resonance adjacent to the primary one, besides compensating the reactive part of the input impedance, changing its value close to zero, making this parameter frequency-independent. This results in an “overlap” between resonances, which broadens the antenna’s frequency response, as demonstrated in [8].

This article presents a four-element MIMO antenna integrated into a low-permittivity substrate. The design incorporates an octagonal SRR-based metamaterial structure to maximize size reduction and a stub in the ground plane to ensure a robust bandwidth covering the n77 and n78 bands. Analysis of diversity metrics (*ECC*, *TARC*, *DG*), along with isolation and *CCL*, confirms the feasibility of the proposal. The design, simulation procedures, and experimental results are detailed in the subsequent sections.

## 2. Unit Cell Design

In [9,10], it was demonstrated that a single pair of SRRs exhibits metamaterial behavior. Consequently, by increasing the number of rings, this behavior is reinforced, and if the rings are embedded inside one another, the effect can be increased over a wider bandwidth, resulting in an enhanced slow-wave factor. This indicates a larger electrical length for a physically smaller structure, enabling highly effective miniaturization.

**Figure 1:**
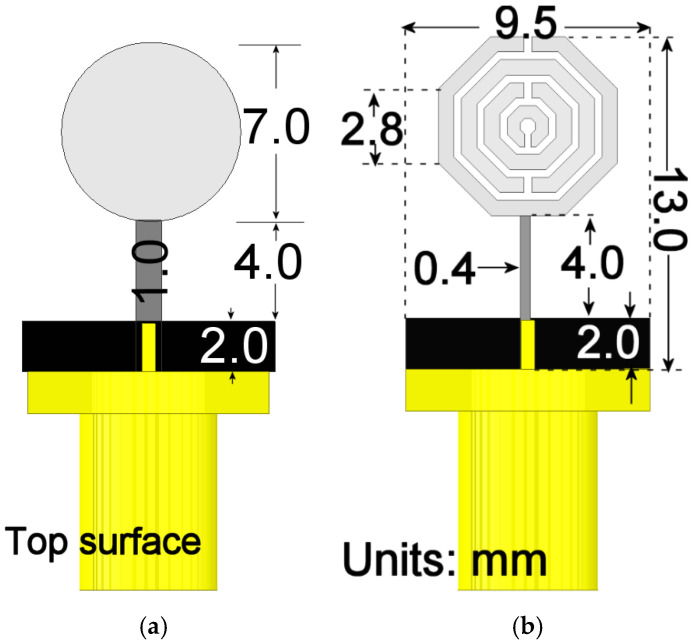
(**a**) Disc monopole and (**b**) MTM-based monopole.

**Figure 2:**
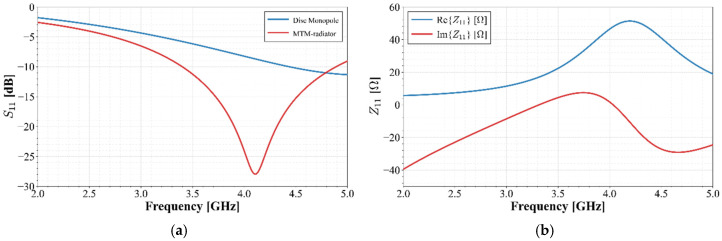
(**a**) $S_{11}$ comparison of disc monopole and MTM-radiator; (**b**) MTM-radiator input impedance behavior.

Based on the size reduction results reported in [3], and for comparison purposes, initial dimensions are proposed for a well-known circular monopole with a 7 mm diameter, as detailed in Figure 1a. The structure is excited by a microstrip line with a width of 1 mm, integrated with a ground plane segment measuring 2 mm by 10 mm, for 50 Ω input impedance. The feedline length is 6 mm. Rogers RT5880 Duroid substrate with a relative permittivity of $\epsilon_{r}=2.2$ and a thickness of *h* = 1.27 mm is proposed. This initial geometry serves as the starting point to achieve a 50% reduction scale at the target frequency of 3 GHz. The frequency response of this configuration is illustrated in Figure 2a.

For the case of the metamaterial-based resonator, the proposed initial design is based on a configuration of two pairs of octagonal split-ring resonators (SRRs), whose dimensions are inscribed on a circular disc of 7 mm. The unit radiator geometry is presented in Figure 1b. The set of SRRs forming the radiator is located on the top layer of the substrate (represented in light gray) and is fed by a microstrip line (shown in dark gray). The ground plane, also implemented with a strip line, is located on the bottom layer of the substrate and is colored black.

The final dimensions of the radiator were determined through iterative optimization using the Ansys Electronics Desktop 2026 simulation environment. The optimization process consisted of modifying both the ring width and the spacing between them, ensuring that the final edge-to-edge distance did not exceed 7 mm to allow for a direct response comparison with the disk monopole. Furthermore, the feedline width was parameterized to enhance impedance matching at the input port. This procedure balanced the competing demands of compact size and extended frequency response while maintaining the matching level below the standard −10 dB; its performance is shown in Figure 2a, where it is compared to the monopole response, and Figure 2b presents its impedance behavior. As observed in Figure 2b, the antenna shows a non-stable reactive part, changing from capacitive to reactive and once again to capacitive behavior between cut-off frequencies. This performance becomes highly frequency-dependent, making it unstable in wideband applications. The optimized unit radiator reaches dimensions of $13\times 9.52\;{mm}^{2}$. The width of each ring is 0.6 mm, except for the central ring, which has a width of 0.5 mm. Furthermore, the inter-ring separation is 0.3 mm.

For this initial operation, it is observed that the resonant frequency of the MTM-based structures is lower than the conventional monopole (around 5 GHz), demonstrating that this structure behaves as expected, increasing the slow-wave factor and shifting the bandwidth to a lower central frequency.

This initial antenna design resonates at a frequency of 4.3 GHz and a bandwidth of 1 GHz, approximately. To contextualize the degree of miniaturization achieved, it is compared to a conventional quarter-wave $\left(\lambda /4\right)$ monopole. At the resonant frequency, a quarter-wave monopole would have a length of 17.44 mm. Therefore, the proposed antenna, with a length of 13 mm, achieves an approximate reduction of 26% relative to this reference, validating the miniaturizing effect of the metamaterial structure.

To confirm that the proposed structure exhibits metamaterial behavior, the analytical procedure described in [11] was followed. This method allows for the extraction of the constitutive parameters (permittivity ϵ and permeability μ) from the scattering parameters. Permittivity is obtained through the relation ϵ=n/z, while permeability is defined as μ=nz, where n represents the refractive index and z is the characteristic impedance of the medium. These parameters are directly linked to the S-parameters via Equations (1)–(4) from [11]:

(1) $$z=\pm \sqrt{\frac{{\left(1+S_{11}\right)}^{2}-S_{21}^{2}}{{\left(1-S_{11}\right)}^{2}-S_{21}^{2}}}$$

(2) $$n=\pm \frac{1}{jL}\left(\frac{c}{\omega }\right)\cosh^{-1}\left(\frac{1-S_{11}^{2}+S_{21}^{2}}{2S_{21}}\right)$$

The signs in (2) and (3) are determined by the following conditions:

(3) $$z^{\prime }\geq 0$$

(4) $$n^{\prime \prime }\geq 0$$

where (·)′ and (·)″ denote the real and imaginary parts of each operator, respectively.

**Figure 3:**
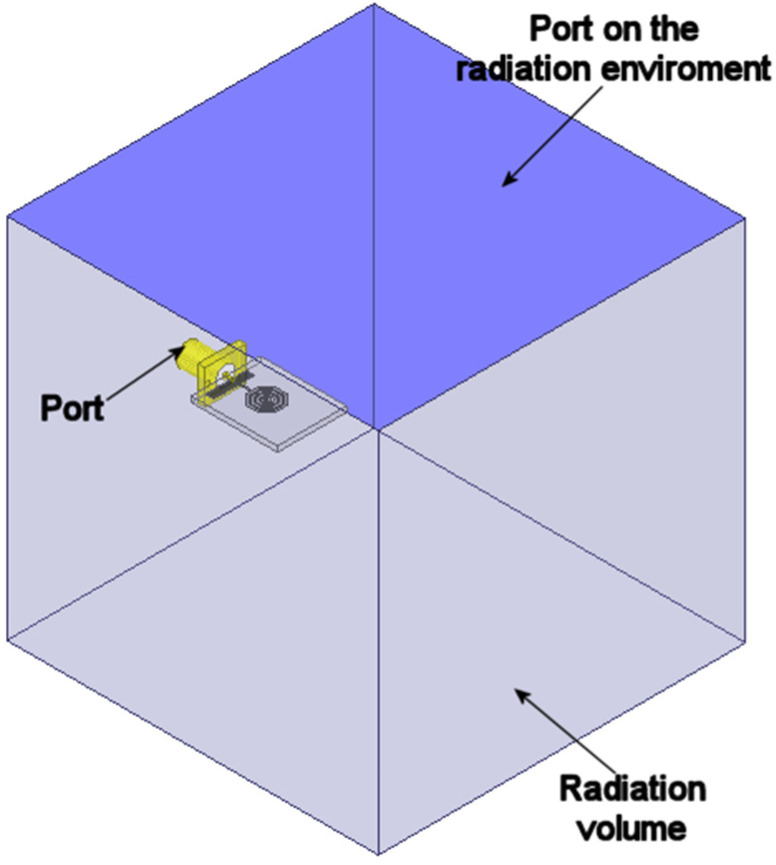
Two-port simulation model.

**Figure 4:**
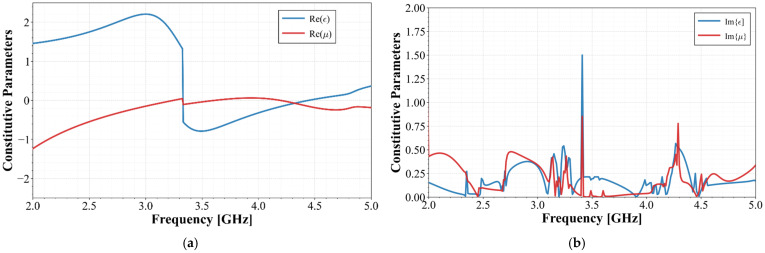
Constitutive parameters; (**a**) real parts of permeability and permittivity; (**b**) imaginary parts of permeability and permittivity.

To obtain the necessary data, the structure in Figure 1 was simulated in a two-port configuration, where the second port was placed on the top face of the radiation boundaries, as shown in Figure 3. Once the data were obtained, they were processed using the aforementioned equations, extracting the real and imaginary parts of the permittivity and permeability, which are presented in Figure 4.

From Figure 4, it is observed that the real part of the retrieved effective permittivity exhibits a sharp Lorentz-type transition from +1.2 to −0.8 at 3.4 GHz, while the imaginary part shows a corresponding peak of 1.5 at the same frequency. This satisfies the Kramers–Kronig causality requirement and confirms that the nested octagonal SRRs behave as a resonant metamaterial near the operating frequency. The negative ε region above 3.4 GHz contributes to the bandgap that makes antenna miniaturization possible. Regarding the retrieved permeability, two narrowband double-negative (DNG) regions are observed, the first one from 3.3 GHz to 3.6 GHz and, the second one, from 4.25 GHz to 4.4 GHz, each with corresponding μ″ peaks (0.77 at 3.4 GHz and 0.76 at 4.28 GHz), confirming real magnetic resonances.

For any structure to be considered an effective metamaterial, the unit cell period *a* must satisfy that *a* << λ, typically *a*/λ < 0.2–0.3. The unit cell shown in Figure 1b has an estimated period *a* ≈ 7.0 mm. At 3.4 GHz, λ_0_ = 88.2 mm; then, a relation of *a*/λ_0_ ≈ 0.079 is obtained, which meets the effective medium condition. However, the *S*-parameter retrieval method given in [11] assumes an isotropic, homogeneous effective medium, whereas the octagonal SRR array presented herein is inherently anisotropic. Consequently, the extracted ε and μ shown in Figure 4 are isotropic scalar approximations valid only for the specific polarization and normal incidence used in this work. A full characterization would require retrieving the full tensor components (ε_xx_, ε_yy_, ε_zz_, μ_xx_, μ_yy_, μ_zz_) and mapping the isofrequency contours to determine whether the structure exhibits elliptical or hyperbolic dispersion. As discussed in [12] for localized resonance structures, alternating DNG/ENG/MNG bands, such as those observed in Table 1, can, in anisotropic implementations, lead to hyperbolic dispersion. A full dispersion analysis is beyond the scope of this antenna-design-focused work, where the extracted parameters serve primarily to demonstrate the resonance frequency shift to a lower value that enables antenna miniaturization.

From Figure 2 and Figure 4, it is clear that the MTM-based radiator allows the resonant frequency of the antenna to be reduced; however, to achieve the goal of covering the n77/n78 band, a wider bandwidth is required with a lower cut-off frequency.

## 3. Ground Plane Stub Implementation and Its Effect on Frequency Response

While the miniaturization technique using octagonal SRRs significantly reduces radiator dimensions, it imposes an inherent limitation: bandwidth reduction. As observed in the previous section, the unit cell exhibited a 1 GHz bandwidth resonating at 4.3 GHz, which is insufficient to cover the bands of interest. To address this, the inclusion of a ground plane stub was explored as a bandwidth enhancement technique.

Several studies have demonstrated that modifying the ground plane with loading structures can improve the frequency response of planar antennas. In [13], a hairpin-shaped monopole antenna is presented where, by connecting an asymmetric T-shaped patch loaded with a horizontal stub to the ground plane, the fractional bandwidth increased from 82% to 164%. Furthermore, the lower operating frequency shifted from 3.6 GHz to 1.95 GHz, evidence that the strategic incorporation of stubs can generate new resonant modes and significantly broaden the frequency response. Similarly, ref. [8] demonstrated the effectiveness of this technique on a modified inverted-L antenna for Wi-Fi applications. By including a ground plane stub, the impedance bandwidth improved from 13.1% to 15.7% in the 2.45 GHz band, and from 10.1% to 19.9% in the 5.2/5.8 GHz bands, doubling the bandwidth at higher frequencies. This underscores the versatility of stubs as loading elements for bandwidth enhancement across various antenna topologies.

**Figure 5:**
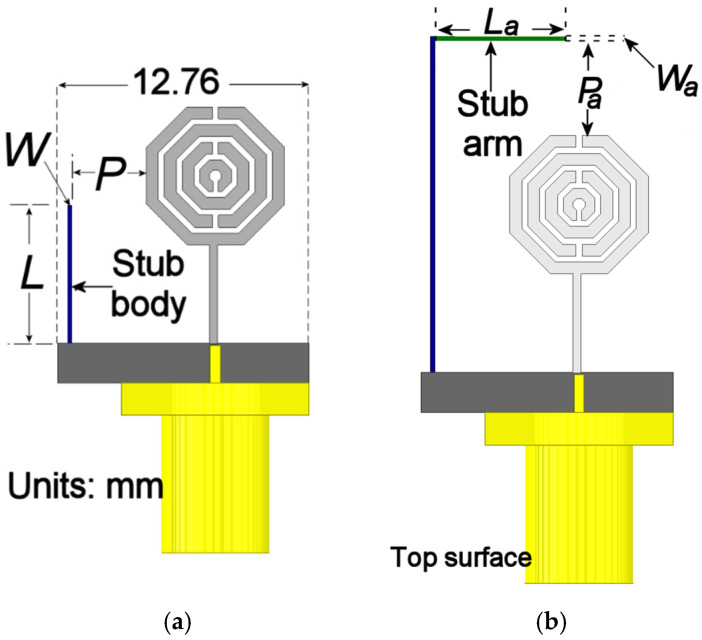
Integration of (**a**) the MTM-radiator with the stub body; (**b**) adding the stub arm (inverted-L configuration).

**Figure 6:**
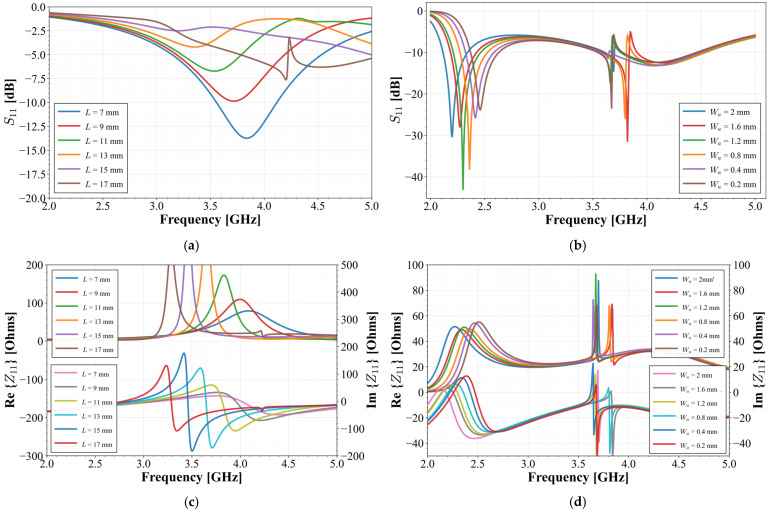
Simulated $S_{11}$ responses for the preliminary design stages: (**a**) the stub length variation; (**b**) the stub arm width variation; (**c**) real and imaginary parts of the input impedance varying L; (**d**) real and imaginary parts of the input impedance varying *W_a_*.

Based on these precedents, a stub was integrated into the ground plane of the proposed unit cell. To provide a larger area for parametric sweeps, the ground plane length was increased to 12.76 mm. The development of the stub structure was carried out in two preliminary stages before the final optimization. First, a vertical stub body was integrated to extend the current path. Subsequently, a horizontal arm was added to introduce a reactive effect and tune the coupling between modes. This last stage is described later. In all stages, three geometric variables were considered: the stub’s length, *L*; width, *W*; and separation from the radiator, *P*. The geometric evolution of these stages is illustrated in Figure 5, while their corresponding impact on the reflection coefficient ($S_{11}$) is compared in Figure 6.

For the initial analysis of stub integration, its length was parameterized. For this analysis, the stub was positioned at an initial offset of *P* = 3.8 mm with a fixed width of W = 0.2 mm. The length L was then swept from 7 mm to 17 mm, and the resulting input response is shown in Figure 6a. It is observed that increasing the stub’s physical length leads to an impedance mismatch at the radiator’s feed point. However, when the stub length is extended to 17 mm, the resonant frequency shifts to a lower value. This frequency shift represents a key design objective. The port coupling will be further enhanced through the implementation of the horizontal stub section, as will be demonstrated in subsequent paragraphs. On the other hand, Figure 6c illustrates the behavior of the radiator’s input impedance. As shown in this figure, increasing the value of *L* results in a reduction in the real part of the impedance within the 3.5 to 5 GHz range, compared to the configuration without the stub (Figure 2b). Furthermore, the reactive part stabilizes over this frequency interval, minimizing its value and approaching zero. This behavior indicates that the stub effectively achieves impedance matching over a broader bandwidth while simultaneously shifting the operating frequency toward the lower frequency spectrum.

Following this initial assessment, the horizontal section of the stub was integrated. A parametric study was conducted on the width of this arm (*W_a_*), which varied from 0.2 mm to 2 mm, while its length (*L_a_*) and position (*P_a_*) were initially set to 10 mm and 4.8 mm, respectively. These dimensions were chosen to ensure it did not exceed the ground plane’s length. The results of this sweep are shown in Figure 6b. As illustrated in this figure, the original resonance continues its downward trend, shifting to approximately 2.2 GHz when the arm width reaches its maximum value. Moreover, the introduction of the horizontal arm excites an additional resonant mode in the 3.5–4 GHz range. This new mode arises from the modified current path along the combined stub structure. Despite the frequency shift achieved, an impedance mismatch at the fundamental resonance persists within the target band.

However, as shown in Figure 6d, the inclusion of the stub arm further stabilizes the input impedance behavior. This modification effectively mitigates the peaks in the real part observed in Figure 6c, maintaining values between 20 and 40 Ohms across most of the target bandwidth. Simultaneously, the imaginary part undergoes compensation, stabilizing within a range from −j40 to 0 Ohms; this contrast is significant compared to the results in Figure 6c, where the reactive values fluctuated between −j300 and j200 Ohms.

According to these results, the following values were selected for each stage: (a) *L* = 17 mm, *W* = 0.2 mm, and *P* = 3.8 mm and (b) *L_a_* = 10 mm, *W_a_* = 0.4 mm.

**Figure 7:**
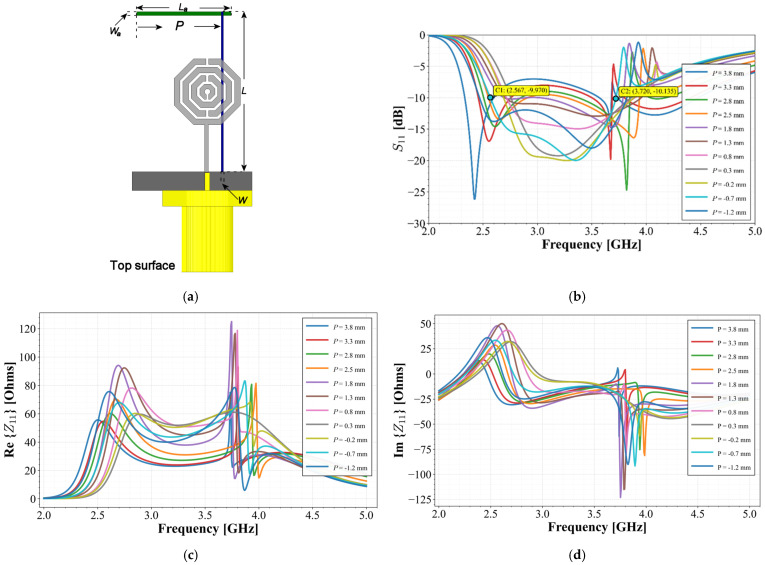
(**a**) Third stage of parametrization configuration; (**b**) *S*_11_ simulated results; (**c**) real part of the input impedance; (**d**) imaginary part of the input impedance.

To achieve the goal of broadening the band and covering the required frequency, a final parametric sweep was performed. The dimensions (*L*, *L_a_*, *W, W_a_* and *P_a_*) were kept constant while varying the relative position of the vertical part of the stub to the radiator. In this case, the distance between the radiator and the stub went to negative coordinates, considering the edge of the SRR as coordinate zero, as depicted in Figure 7a. Figure 7b displays the results of this parametrization.

As observed in Figure 7b, the frequency response of the radiator is significantly improved, covering the required band when position P assumes negative values. In this case, the vertical arm of the stub is positioned below the main radiator, achieving a trade-off between bandwidth and port coupling depth. For this configuration, a value of *P* = −0.7 mm was adopted, resulting in a bandwidth spanning from 2.56 GHz to 3.72 GHz, while the *S*_11_ parameter reaches values close to −20 dB. Within this bandwidth, it is observed that for a value of *P* = −0.7 mm, the real part of the impedance remains between 45 and 65 Ohms, while the imaginary part remains stable at approximately -j10 Ohms. This implies that the stub enhances the impedance characteristics by matching the real part close to the required 50 Ohms and shifting the imaginary part toward a negligible value near 0 Ohms. Furthermore, the stub maintains these values over a wide frequency range, resulting in a frequency-independent behavior for the antenna, as displayed in Figure 7c,d.

**Figure 8:**
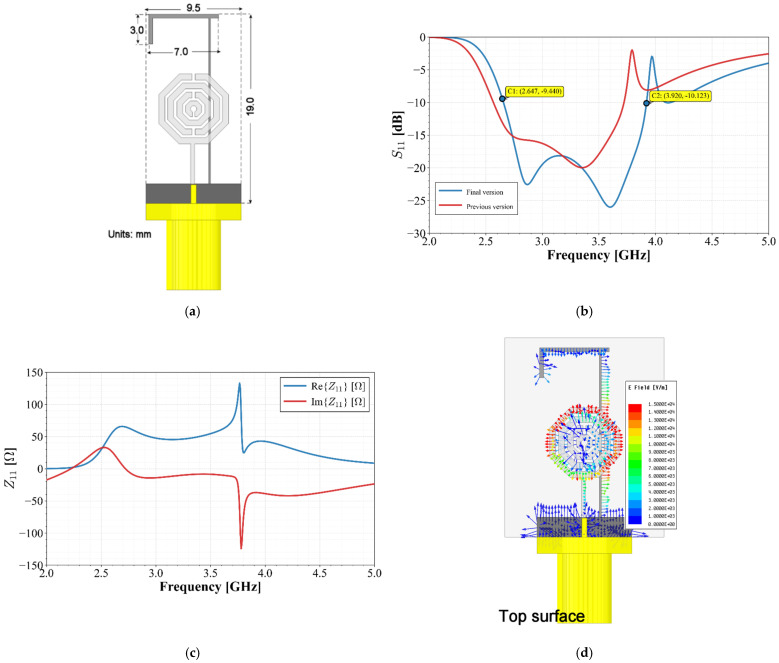
(**a**) Optimized unit radiator final design; (**b**) S11 parameter; (**c**) input impedance; (**d**) electric field distribution at the middle band.

This design achieved dimensions of $19\times 12.76\;{mm}^{2}$ and a bandwidth from 2.56 GHz to 3.72 GHz. To reach extreme miniaturization, the ground plane width was reduced to the minimum required for an SMA connector, and a fold was added to the stub arm to keep it within the ground plane width. Figure 8a shows the final geometry and Figure 8b presents a comparison of the $S_{11}$ response before and after these modifications.

These adjustments do not significantly affect performance but improve the bandwidth at the higher cut-off frequency. The final design reaches dimensions of $19\times 9.52\;{mm}^{2}$, maintaining a bandwidth from 2.6 GHz to 3.9 GHz, a 30% improvement over the initial stub-less design. The input impedance, as observed in Figure 8c, shows how the use of the stub stabilizes this parameter, changing the real part to a value close to 50 Ohms and keeping the reactive part steady and close to zero Ohms (less than -j10 Ohms), thereby maintaining its characteristic of being nearly frequency-independent all over the operating bandwidth. Furthermore, Figure 8d illustrates the behavior of the electric field vector at the center frequency. It can be observed that the field is induced from the radiator toward the stub and subsequently returns to the ground plane. This mechanism increases the electrical length traversed by the electric field, thereby shifting the antenna’s response toward lower frequencies and resulting in a reduction in the radiator’s physical size.

**Figure 9:**
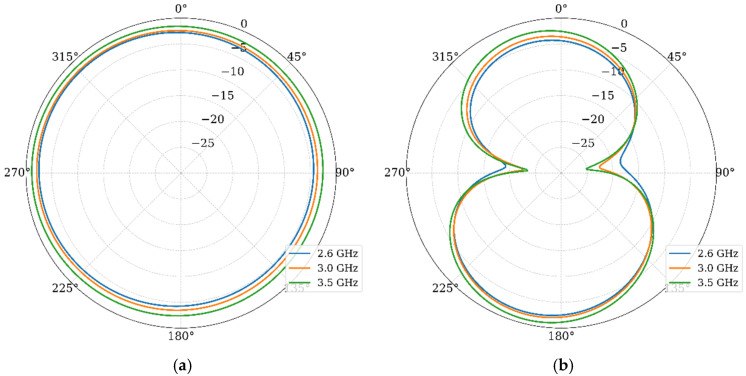
Normalized radiation patterns: (**a**) H-plane and (**b**) E-plane.

Finally, the radiation patterns were analyzed. Figure 9 presents the normalized gain patterns in the E and H planes at 2.6 GHz, 3 GHz, and 3.5 GHz.

In the E-plane, the pattern shows a modified monopole characteristic with slight asymmetry due to the stub. In the H-plane, the pattern is quasi-omnidirectional, which is ideal for mobile terminals. The radiation pattern remains stable across the band, indicating that the primary radiation mechanism is preserved despite the bandwidth enhancement. The simulated peak realized gains and the radiation efficiency are −2.2 dBi, −1.8 dBi, and −0.8 dBi and 98%, 98%, and 99% for 2.6 GHz, 3.0 GHz, and 3.5 GHz, respectively. These values are very well expected, since the reduction in the antenna size directly impacts the efficiency gain-bandwidth. In this case, the bandwidth is not only preserved, but increased, then the gain is slightly reduced. On the other hand, since the substrate has a very low loss tangent of 0.0004 and the structures are very small, the antenna presents low losses, giving a higher radiation efficiency.

Once the unit radiator’s behavior was optimized, validating its bandwidth, miniaturization, and radiation patterns, the study proceeded to integrate four of these elements into a MIMO configuration, arranging the elements in an axial symmetry. The design, spatial arrangement, and performance analysis of this array are presented in the following section.

## 4. Four-Element MIMO Array Configuration

Once the final unit radiator design was established, four elements were integrated into a compact MIMO configuration. The radiators were arranged in an axial symmetry to obtain an orthogonal orientation, imposing a 90° rotation between adjacent elements. This layout provides intrinsic polarization diversity, which contributes to mutual isolation.

**Figure 10:**
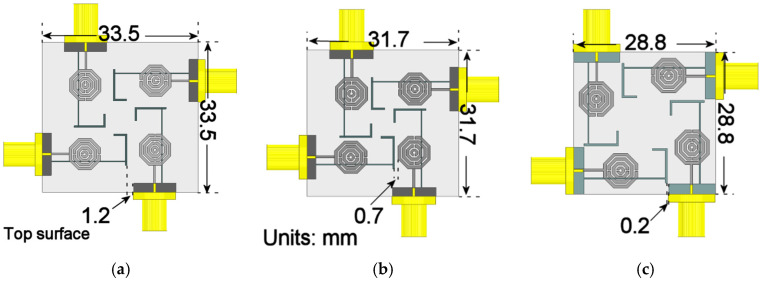
Evolution of the MIMO array configuration: (**a**) Stage 1, *d* = 1.2 mm, stubs facing adjacent radiators; (**b**) Stage 2, *d*= 0.7 mm; (**c**) Final stage, *d* = 0.2 mm, stubs facing adjacent ground planes.

**Figure 11:**
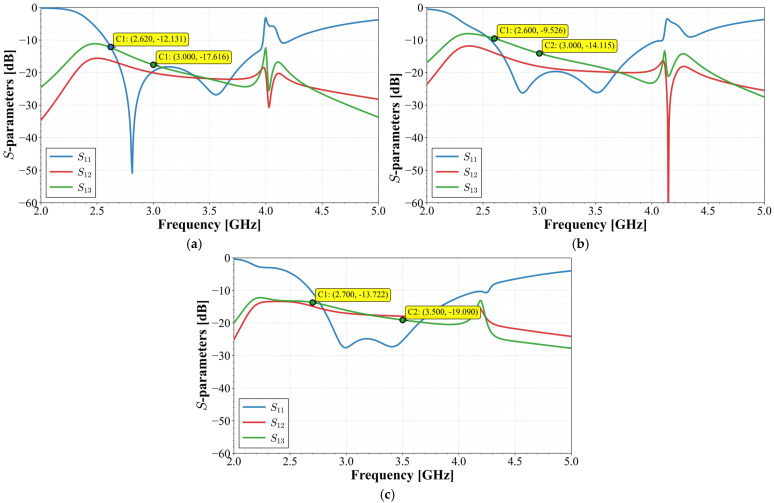
Simulated S-parameter performance at each stage of the design evolution: (**a**) Stage 1; (**b**) Stage 2; (**c**) Final stage.

However, the high integration density required for mobile terminal applications necessitates extreme proximity between elements, which can degrade isolation. To determine the optimal configuration that maximizes isolation without compromising compactness, the design process was evaluated in three stages involving refinements in inter-element separation (*d*) and stub orientation. The geometric evolution of these stages is illustrated in Figure 10, while their corresponding scattering parameters are compared in Figure 11.

As observed in Figure 11, the main disadvantage in the three configurations is the isolation in the lower band. Stage 1 shows an isolation level of 12 dB, while Stage 2 and 3 present an isolation of −9.5 dB and −13.7 dB, respectively. However, as the operating frequency increases, the isolation between elements also improves, reaching values exceeding 15 dB starting from 3 GHz, and approaching 20 dB near the upper cut-off frequency

**Figure 12:**
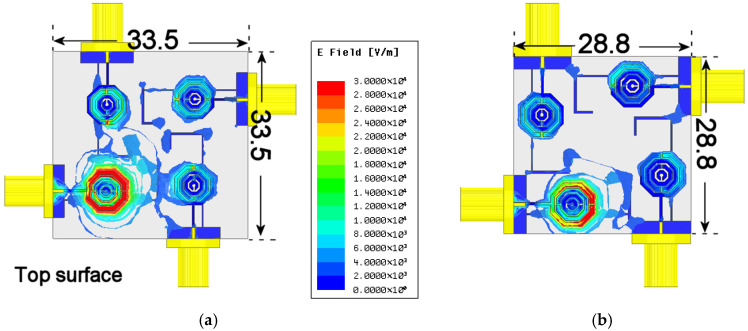
Surface current distribution comparison: (**a**) Stage 1, showing strong coupling between the stub and the adjacent radiator; (**b**) Final stage, showing the ground plane absorbing the radiated energy from the stub.

To demonstrate the isolation, taking into account only the first and third stages, an electric-field distribution analysis is carried out at the middle band. As shown in Figure 12a, the electric field intensity is more prominently induced in the adjacent radiators, as evidenced by the shift toward green and red hues across a significant portion of the metamaterial structure. This contrasts with the results observed in Stage 3, depicted in Figure 12b, where the induction levels are noticeably lower, characterized by colors closer to the blue spectrum. These results demonstrate that the electromagnetic interaction between the radiating elements of the array is considerably weaker in the final stage compared to the initial one.

This final stage allows the complete array to reach total dimensions of 28.8×28.8 mm^2^, equivalent to $0.24\lambda_{0}\times 0.24\lambda_{0}$ at the lower cut-off frequency of 2.6 GHz, while ensuring high port isolation. Despite a physical separation of 0.2 mm (equivalent to 0.0017$\lambda_{0}$) between the radiating elements, this high degree of isolation is maintained.

**Figure 13:**
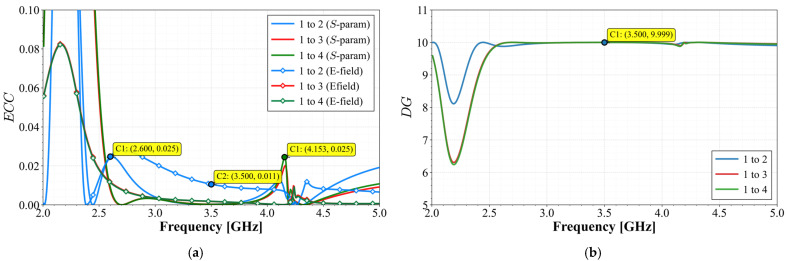
Calculated diversity performance for Antenna 1 and the other elements: (**a**) *ECC* comparison with *S*-parameters and E-field methods and (**b**) Diversity Gain.

The Envelope Correlation Coefficient (*ECC*) was calculated for each antenna pair. Due to the axial symmetry, the analysis focuses on the *ECC* between Antenna 1 and the remaining radiators. According to (5), the *ECC* is obtained by calculating the electric field over the sphere. However, this method is complicated and hard to achieve, especially in the laboratory. Because of this drawback, for high efficiency antennas, a derivation of the *ECC* based on the *S*-parameters can be applied when the radiator achieves an efficiency above 90%, and this expression is given in (6). As demonstrated above, this array achieves a radiation efficiency clearly above 90%; then, using (5) and (6), the results are given in Figure 13a. The E-field method was calculated by employing the Ansys MIMO Toolkit.

(5) $$ECC=\frac{{\left|\iint_{4\pi }\left[{\vec{E}}_{i}\left(\theta ,\varphi \right)•{\vec{E}}_{j}\left(\theta ,\varphi \right)\right]d\Omega \right|}^{2}}{\iint_{4\pi }{\left|{\vec{E}}_{i}\left(\theta ,\varphi \right)\right|}^{2}d\Omega \iint_{4\pi }{\left|{\vec{E}}_{j}\left(\theta ,\varphi \right)\right|}^{2}d\Omega }$$

where ${\vec{E}}_{k}\left(\theta ,\varphi \right)$ is a complex vector function that describes the *k*th 3D radiation pattern at the Fraunhofer zone, • is the Hermitian product, and dΩ is the solid angle differential.

(6) $$ECC=\frac{{\left|S_{11}^{*}S_{12}+S_{21}^{*}S_{22}\right|}^{2}}{\left(1-{\left|S_{11}\right|}^{2}-{\left|S_{21}\right|}^{2}\right)\left(1-{\left|S_{22}\right|}^{2}-{\left|S_{12}\right|}^{2}\right)}$$

Complementarily, the Diversity Gain (*DG*) was evaluated using Equation (7):

(7) $$DG=10\sqrt{1-{\left|ECC\right|}^{2}}$$

The results reveal that the *ECC*, with both methods, presents great convergence, reaching values below 0.03 at the start of the band and drops significantly below 0.01 across the rest of the frequency range, indicating an extremely low correlation level. The analysis for the *DG* displayed in Figure 13b shows that the system reaches values close to the theoretical maximum, achieving a value of 9.999 at the middle band and almost across all the bands, ensuring efficient multipath fading mitigation.

Another very important metric to determine the efficiency of the MIMO array is the Total Active Reflection Coefficient (*TARC*). This metric allows one to determine the total system bandwidth in a multipath environment, where multiple trajectories can arrive at the array with different phases and delays. This metric was evaluated to determine the effective bandwidth under simultaneous operation according to Equation (8):

(8) $$TARC=\sqrt{\frac{\sum_{i=1}^{N}{\left|b_{i}\right|}^{2}}{\sum_{i=1}^{N}{\left|a_{i}\right|}^{2}}}=\frac{1}{\sqrt{N}}\sqrt{\sum_{i=1}^{N}{\left|\sum_{k=1}^{N}S_{ik}e^{j\theta_{k-1}}\right|}^{2}}$$

**Figure 14:**
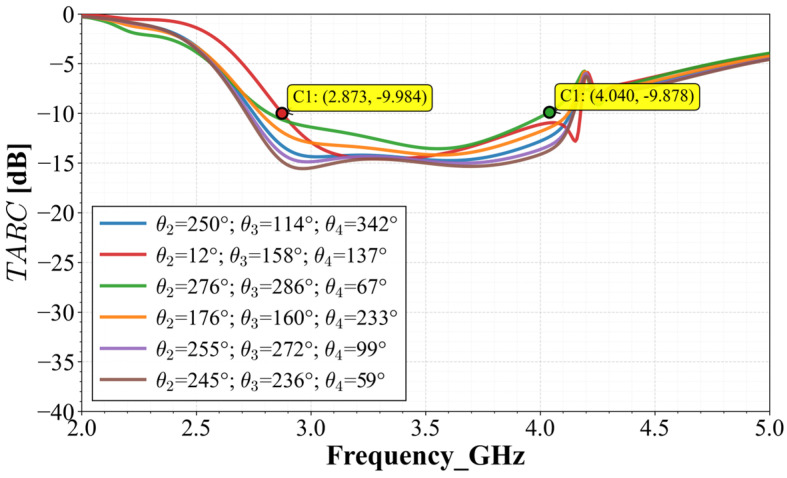
Calculated *TARC* for six random phase combinations.

The results of this operation are presented in Figure 14. As shown in this figure, the curves for various incident random phases exhibit a convergent pattern, maintaining the system bandwidth below −10 dB from 2.8 GHz to 4 GHz, comfortably covering the n77 and n78 bands.

Channel Capacity Loss (*CCL*) was also evaluated to ensure that the array does not significantly degrade system capacity. This loss is assessed using Equation (9):

(9) $$CCL=-log_{2}\left(\left|\Psi^{R}\right|\right)$$

where $\left|\cdot \right|$ denotes the determinant operator, and $\Psi^{R}$ represents the correlation matrix of the receiving antenna. The correlation matrix can, in turn, be evaluated using Equation (10):

(10) $$\Psi^{R}=\left[\begin{array}{ccc} \rho_{ii} & \rho_{ij} & \rho_{ij}\\ \rho_{ij} & \rho_{ii} & \rho_{ij}\\ \rho_{ij} & \rho_{ij} & \rho_{ii} \end{array}\right]$$

where

(11) $$\rho_{ii}=1-\sum_{n=1}^{4}\left|S_{in}^{*}S_{ni}\right|$$

(12) $$\rho_{ij}=-\sum_{n=1}^{4}S_{in}^{*}S_{nj}\;para\;i\neq j$$

**Figure 15:**
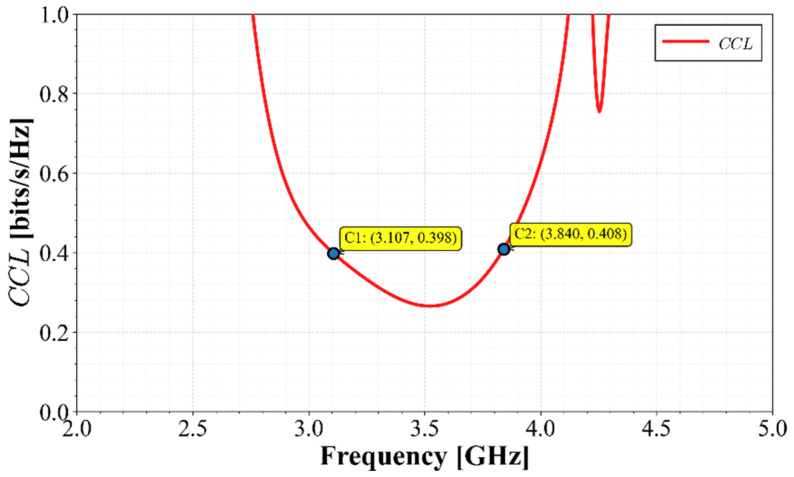
Calculated *CCL* of the MIMO array.

Evaluating (9) to (12), the results are presented in Figure 15. As seen in this figure, the *CCL* does not exceed the recommended threshold of 0.4 bits/s/Hz between 3.1 GHz and 3.8 GHz, implying that the array maintains high channel efficiency.

**Figure 16:**
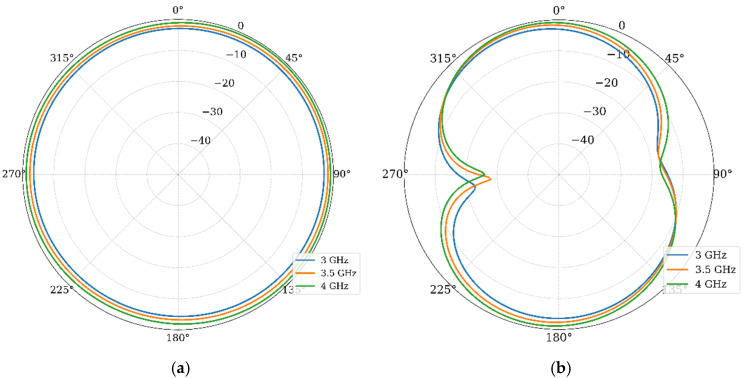
Normalized gain patterns of the MIMO array at 3 GHz, 3.5 GHz, and 4 GHz: (**a**) H-plane and (**b**) E-plane.

Finally, the simulated gain patterns are given in Figure 16. These results show that the array maintains omnidirectional coverage in the azimuthal plane. This confirms that the polarization diversity introduced by the orthogonal orientation does not compromise global coverage. These results, combined with the diversity metrics, confirm the suitability of the proposed design for 5G MIMO applications in compact devices.

The radiation results, in conjunction with the diversity metrics presented, confirm the suitability of the proposed design for MIMO applications in compact 5G communication devices, demonstrating stable and consistent behavior across the entire operating band. These findings are further validated through the experimental results and comparative analysis presented in the following sections.

## 5. Construction and Characterization of the MIMO Array

**Figure 17:**
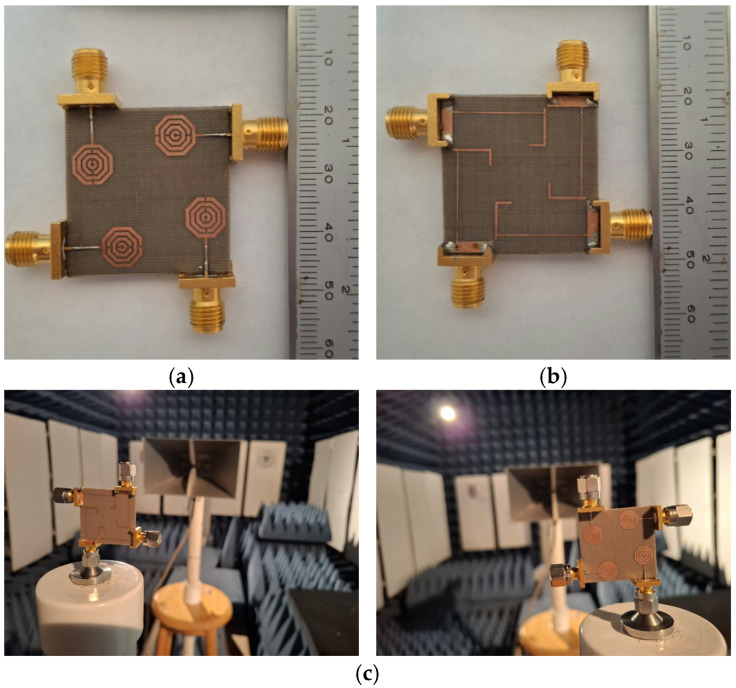
Prototype of the 4-element MIMO antenna: (**a**) front view, (**b**) back view, and (**c**) interior of the anechoic chamber.

Once the prototype was fabricated, its performance was experimentally characterized. Figure 17 shows the fabricated array along with its setup within the anechoic chamber for radiation pattern measurements.

**Figure 18:**
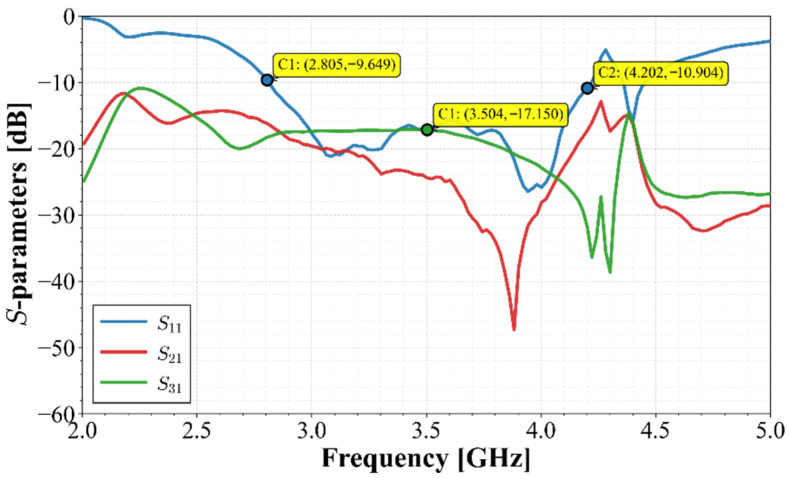
Measured S-parameters.

Figure 18 presents the measured scattering parameters of the array. Due to the design symmetry, the reflection coefficients of the four ports are nearly identical; thus, only $S_{11}$ is shown as a representative plot for display clarity. Regarding isolation, the curves corresponding to couplings with Port 1 ($S_{21},S_{31}$) are presented, as the remaining combinations are equivalent due to the geometric arrangement.

The results, given in Figure 18, show that the array maintains good impedance matching in the band of interest, with $S_{11}<-10\;dB$ ranging from 2.8 GHz to 4.2 GHz, slightly lower than the simulated results, still covering the required 5G bands. This discrepancy is attributable to manufacturing tolerances and additional losses introduced by the SMA connectors. Regarding isolation, both cases exceed the 15 dB reference value, remaining stable across the band and achieving peaks above 30 dB at the higher cut-off frequency. These results confirm that the strategy of orienting stubs toward neighboring ground planes remains effective in the fabricated prototype.

**Figure 19:**
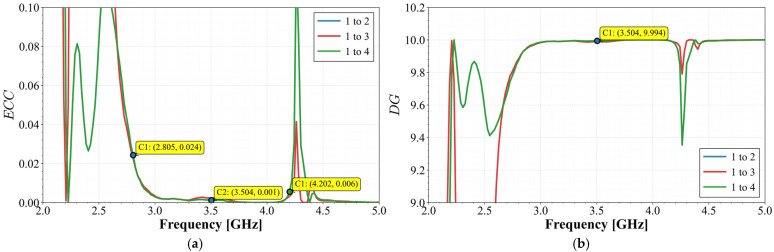
Calculated diversity performance between ports: (**a**) *ECC* and (**b**) *DG*.

From the measured *S*-parameters, the MIMO diversity metrics were calculated. Figure 19 shows the Envelope Correlation Coefficient (*ECC*) and the Diversity Gain (*DG*) between Port 1 and the other elements.

The *ECC* values remain below 0.005 throughout the entire operating band. This exceptionally low correlation level reflects the effectiveness of the orthogonal element arrangement combined with the stub orientation strategy. The *DG* values remain above 9.99 for all pairs across the band, approaching the theoretical limit. This indicates that the array efficiently exploits both spatial and polarization diversity.

**Figure 20:**
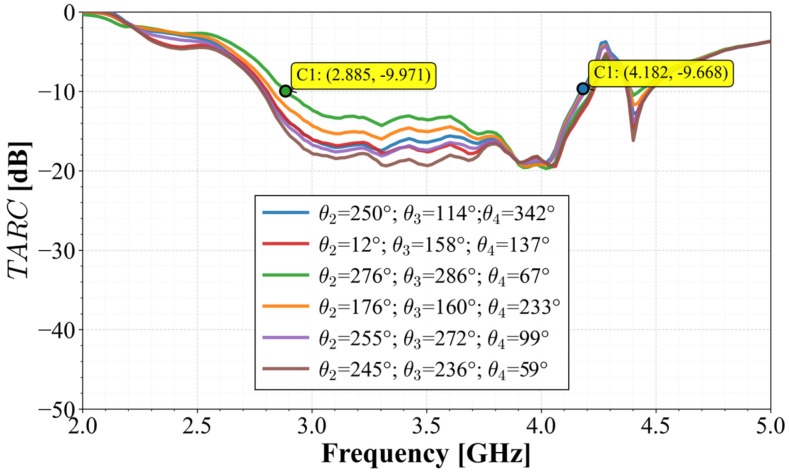
Calculated *TARC* for six sets of random phase combinations.

One of the most demanding metrics for MIMO systems is the Total Active Reflection Coefficient (*TARC*), as it considers the array’s behavior when all ports are simultaneously excited with arbitrary phases. Using six sets of random phase combinations, the measured S-parameters were processed to obtain the curves shown in Figure 20.

The resulting curves exhibit remarkable convergence, staying below −10 dB across the 2.8 GHz (C1) to 4.1 GHz (C2) range. This demonstrates that, regardless of the relative phases of the incident signals, the system maintains its efficiency and stability.

**Figure 21:**
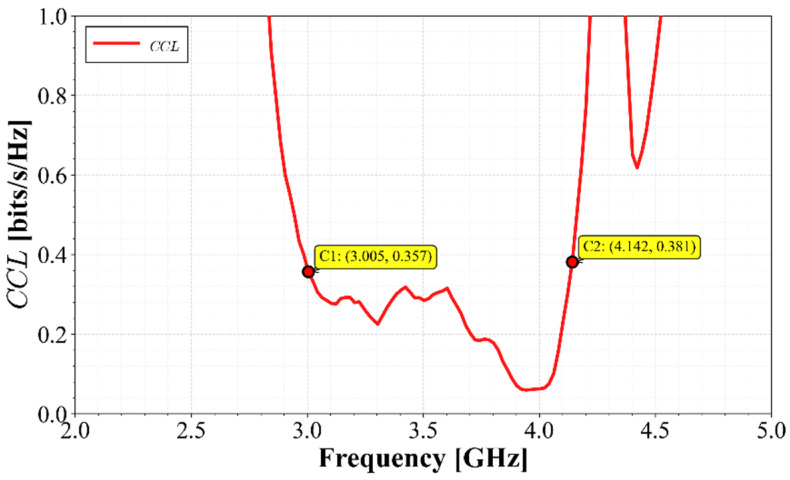
Calculated Channel Capacity Loss (*CCL*).

The Channel Capacity Loss (*CCL*) was evaluated by applying Equations (8)–(11) to the measured data. As shown in Figure 21, the *CCL* remains below the recommended threshold of 0.4 bits/s/Hz across the entire operating bandwidth, confirming that inter-element coupling does not degrade the MIMO channel capacity.

**Figure 22:**
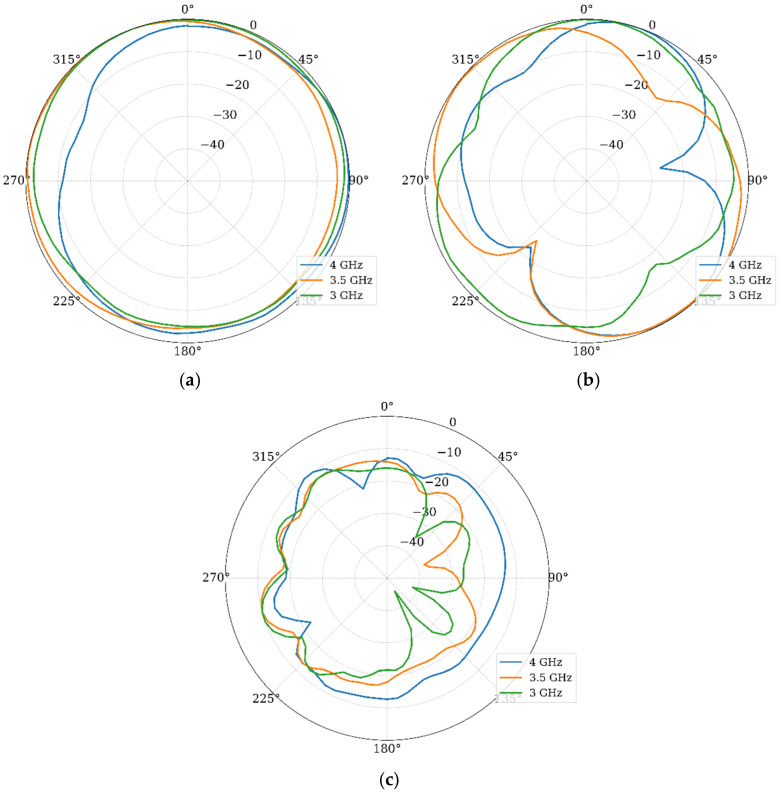
Measured radiation patterns: (**a**) H-plane, (**b**) E-plane, and (**c**) cross-polarization.

Figure 22 displays the normalized gain patterns measured for Port 1 at 3 GHz, 3.5 GHz, and 4 GHz, including both E and H planes and the cross-polarization levels.

In the H plane, the measured patterns confirm the quasi-omnidirectional behavior predicted in simulations, while the E plane presents the well-known nulls forming an 8-shaped pattern. General agreement is observed, with minor differences in pattern uniformity attributable to fabrication tolerances, SMA connector effects, and experimental setup imperfections. The measured cross-polarization levels remain at least 10 to 15 dB below the co-polarization in the direction of maximum radiation, consistent with simulated values. This polarization purity is suitable for MIMO systems, where polarization discrimination contributes to effective channel isolation. Finally, the measured gain values for those frequencies are −1.67 dBi, −0.92 dBi, and −0.3 dBi, agreeing with the simulated results.

The strong correlation between simulation and measurement validates both the electromagnetic model and the fabrication process. To contextualize the performance of the proposed array, Table 2 presents a comparison with state-of-the-art compact MIMO antennas.

From Table 2, it is noteworthy that the proposed design achieves one of the most compact footprints while maintaining metrics comparable to the best works in the literature. For a fair comparison, the lower cut-off frequency in each case was considered to determine the wavelength in free space and to scale the dimensions according to $\lambda_{0}$. Furthermore, unlike some references that omit *TARC*, this work presents an analysis with six completely random phase combinations, demonstrating the design’s robustness under real operating conditions. It is noteworthy that the work presented in [17] is a similar structure; however, the geometry in that work is achieved with double the number of rings and a bigger area, leading to a lesser size reduction, besides needing electromagnetic barriers to improve the inter-radiator isolations, which this work does not require, resulting in an improvement in the design.

## 6. Discussion of Results

The proposed configuration, which combines a structure emulating metamaterial (MTM) behavior, under perpendicular excitation and propagation directions, with an open-ended stub to enhance antenna performance, offers two primary advantages over state-of-the-art radiators. First, the integration of the MTM structure enables a significant reduction in the resonant frequency compared to a conventional quarter-wavelength monopole of equivalent physical length, thereby achieving a more compact footprint for lower operating frequencies. Second, the inclusion of the stub broadens the impedance bandwidth and further shifts the lower cut-off frequency, resulting in both enhanced bandwidth and frequency miniaturization. An analysis of the results presented in Figure 2a and Figure 8b demonstrates that the combination of these two miniaturization techniques shifts the radiator’s operating frequency from 4.5 GHz to 2.6 GHz (based on the lower cut-off frequency) relative to a standard *λ*/4 monopole. Furthermore, the impedance matching was improved; the real part of the input impedance was brought closer to 50 Ohms, while the imaginary part was minimized and remained stable near j0 Ohms. This results in frequency-independent behavior within the operating band, which was expanded from 1 GHz to 1.4 GHz without significantly compromising radiation efficiency, which remains above 90% at the center frequency. Moreover, when implementing a four-element axial array, the broadband characteristics (spanning from 2.6 GHz to 4.3 GHz) were preserved, exhibiting remarkably low Envelope Correlation Coefficients (*ECCs*) and high inter-port isolation, despite the minimal separation between radiators. However, the trade-off for this miniaturization is a reduction in realized gain, with values of approximately −1 dBi at the lower end of the band and exceeding 1 dBi at the upper end.

## 7. Conclusions

This paper presents the design, simulation, fabrication, and characterization of a compact four-element MIMO antenna based on an octagonal SRR metamaterial structure. The design successfully integrates two key strategies to achieve an optimal balance between miniaturization and high-level performance: the use of octagonal SRRs to confine electromagnetic fields within sub-wavelength volumes, and a ground-plane stub-loading network to enhance the frequency response.

Despite an exceptionally reduced inter-element spacing of only 0.2 mm, the array exhibits an isolation exceeding 15 dB without the need for additional decoupling structures. This result was achieved through a novel stub-orientation strategy, where stubs are directed toward neighboring ground planes, allowing the ground to absorb a portion of the radiated energy and significantly mitigate mutual coupling.

The diversity metrics obtained meet the stringent requirements for modern MIMO applications, with an *ECC* of less than 0.005, a Diversity Gain (*DG*) exceeding 9.99, and a *TARC* below −10 dB under multiple random phase combinations. Furthermore, the *CCL* remained below 0.4 bits/s/Hz across the entire operating band. Measured radiation patterns confirmed stable, quasi-omnidirectional coverage with cross-polarization levels well below the co-polarization threshold.

The proposed design achieves its primary objective: a highly compact MIMO antenna array (0.25λ_0_ × 0.25λ_0_) that fulfills all diversity and performance requirements for 5G applications in the n77 and n78 bands. Its simple fabrication process and robust performance make it an ideal candidate for integration into the next generation of compact 5G communication devices.

## Figures and Tables

**Table 1:** Summary of constitutive parameter extraction.

Parameter	First DNG Band	Second DNG Band
Frequency range	3.3–3.6 GHz	4.25–4.4 GHz
ε′ (approx.)	−0.8	Negative (smaller magnitude)
μ′ (approx.)	−0.15	Negative
ε″ peak	1.5 at 3.4 GHz	0.55 at 4.28 GHz
μ″ peak	0.77 at 3.4 GHz	0.76 at 4.28 GHz
Causality satisfied	Yes	Yes

**Table 2:** Comparison of the current work with state-of-the-art elements.

Ref.	# of Ports	Size (${\lambda_{0}}^{2}$)	Bandwidth [GHz]	$S_{ij}$ [dB]	*ECC*	*DG*	*TARC* [dB]	*CCL* [bits/s/Hz]
[2]	4	$0.36\lambda_{0}\times 0.36\lambda_{0}$	3.14–11.23	>20	<0.35	~10	<−10	<0.4
[3]	8	$0.53\lambda_{0}\times 0.53\lambda_{0}$	3–4	>40	<0.0001	~20 dB	<−10	NA
[4]	4	$1.08\lambda_{0}\times 1.08\lambda_{0}$	3–5	>33	<0.17	9.9	NA	<0.4
[9]	8	$0.487\lambda_{0}\times 0.487\lambda_{0}$	3.25–3.95	>20	<0.05	~20 dB	<−15	NA
[14]	2	$1.47\lambda_{0}\times 1.47\lambda_{0}$	8–12	>35	<0.0025	9.99	<−10	~0.3
[15]	4	${0.3\lambda }_{0}\times 0.3\lambda_{0}$	2–10.6	>17	<0.005	NA	NA	<0.3
[16]	2	$1.1\lambda_{0}\times 0.825\lambda_{0}$	27.5–28.5	>30	~0	~10	NA	NA
[17]	4	$0.39\lambda_{0}\times 0.39\lambda_{0}$	3–4.1	>20	<8 × 10^−5^	~10	<−10	NA
This work	4	$0.25\lambda_{0}\times 0.25\lambda_{0}$	2.8–4.2	>30	<0.005	9.9	<−10	<0.4

## Data Availability

The original contributions presented in this study are included in the article. Further inquiries can be directed to the corresponding author.
